# Screening and Efficacy Evaluation of Deodorizing Microbes for Manure Waste in Cold Regions: A Case Study of Xinjiang, China

**DOI:** 10.3390/microorganisms14071548

**Published:** 2026-07-15

**Authors:** Tao He, Wenya Zhang, Xinyi Ren, Bo Yang, Jinliang Sheng, Yu Pan

**Affiliations:** 1 Xinjiang Production and Construction Corps Key Laboratory for Efficient Utilization of Non-Grain Feed Resources, International Joint Research Center for Healthy Animal Farming, College of Animal Science and Technology, Shihezi University, Shihezi 832003, China; ht960704@163.com (T.H.); zhangwyshzu@163.com (W.Z.); 18071743215@163.com (X.R.); 18089337984@163.com (B.Y.); 2 National Risk Assessment Laboratory for Antimicrobial Resistance of Microorganisms in Animals, College of Veterinary Medicine, South China Agricultural University, Guangzhou 510642, China

**Keywords:** cold regions, deodorizing microorganisms, ammonia, hydrogen sulfide

## Abstract

Conventional deodorizing microbial agents exhibit poor cold tolerance. This study aimed to obtain functional strains capable of degrading ammonia and hydrogen sulfide by screening odor-polluted environments in multiple locations in Xinjiang, China. Deodorizing bacteria were isolated using selective media. Their deodorization efficiency was determined using the zinc ammonium complex salt colorimetric absorption method and the boric acid absorption Kjeldahl nitrogen determination method. The strains were identified through 16S rRNA gene sequence analysis. The obtained strains were then formulated into a deodorizing microbial agent, and their combined deodorization effect was evaluated. Three ammonia-degrading strains were obtained, identified as *Serratia proteamaculans*, *Glutamicibacter bergeri*, and *Shewanella putrefaciens*, with maximum degradation rates reaching 52.62%, 63.81%, and 50.80%, respectively, on day 10. Additionally, two hydrogen sulfide-degrading bacteria belonging to the genus Pseudomonas, designated N16 and N56, were isolated under low-temperature conditions. Strain N16 achieved its highest degradation rate of 43.52% on day 5, while strain N56 reached 57.50% on day 10. A highly efficient deodorizing microbial agent, designated Agent E, was ultimately obtained, exhibiting an ammonia degradation rate of 52.67% and a hydrogen sulfide degradation rate of 78.74%.

## 1. Introduction

The livestock industry is a core supplier of animal protein [1]. In recent years, as breeding scale has continued to expand alongside economic development and urbanization, odor pollution from livestock farming has attracted increasing public concern [2]. Common deodorization methods include physical, chemical, and microbial approaches. Among these, microbial deodorization has become the preferred method for livestock farms due to its advantages of safety, environmental friendliness, and cost-effectiveness [3,4]. This method decomposes odor-causing substances such as ammonia, hydrogen sulfide, and volatile fatty acids into harmless or low-toxicity products, including carbon dioxide, water, and nitrates, offering high efficiency, safety, and low cost [5,6,7]. However, the deodorizing efficiency of conventional microbial agents declines sharply when temperatures drop, as most commercial inoculants are mesophilic and lose metabolic activity at suboptimal temperatures [6]. In practical livestock operations, although outdoor winter temperatures in cold regions such as Xinjiang can fall below −20 °C, the temperatures within animal houses are substantially moderated by collective animal body heat and insulation, typically remaining between 10 and 15 °C during winter months [8]. This operational temperature range, while moderate compared to ambient conditions, still falls near the lower functional limit of conventional deodorizing agents, leading to reduced treatment performance during winter. Therefore, screening microorganisms that can maintain deodorizing activity at this typical winter operational temperature (approximately 15 °C) is of practical significance for improving year-round biological treatment in cold-region livestock farms [9].

Low-temperature-deodorizing bacteria, particularly psychrophiles, can maintain high metabolic activity and degradation capacity in cold environments. Xiaojun Jia demonstrated that low-temperature bacteria not only initiate the composting process under low-temperature conditions but also extend the thermophilic phase, thereby improving composting efficiency [10]. These microorganisms can effectively degrade organic pollutants at low temperatures and produce cold-active enzymes that further accelerate pollutant decomposition [11]. Tian XP et al. successfully implemented composting of mushroom residue and wood chips under low temperature by inoculating a psychrotolerant cellulolytic microbial agent, with the composting entering the thermophilic stage on the third day and achieving complete maturation after 84 days [12]. Studies have shown that certain low-temperature microorganisms exhibit excellent degradation performance toward both high- and low-molecular-weight environmental pollutants, such as proteins and carbohydrates [13]. A recent study found that a biochar-immobilized psychrotrophic bacterial consortium achieved 73.9% hemicellulose and 62.4% cellulose degradation within 90 days at 10 ± 2 °C while reducing heavy metals and improving compost maturity and plant growth [14]. These findings demonstrate the strong adaptability and pollution remediation potential of low-temperature microorganisms in cold environments.

Given the practical temperature constraints of livestock houses in Xinjiang during winter and the urgent need for effective odor control, this study aimed to screen functional microorganisms capable of maintaining high deodorizing activity at 15 °C—a representative winter operational temperature for in-house bio-treatment systems. By systematically evaluating their ammonia and hydrogen sulfide removal efficiency at this temperature, this research provides a feasible solution to extend the effective deodorization period of biological treatment systems during the cold months in seasonally cold regions.

## 2. Materials and Methods

### 2.1. Sample Collection

A total of 12 samples were collected from five livestock farms located in Northern Xinjiang, China, including one pig farm, two cattle farms, one poultry farm, and one sheep farm. Samples comprised fresh feces (*n* = 3), compost (*n* = 3), malodorous soil (*n* = 1), wastewater (*n* = 4), and sludge (*n* = 1), collected from the vicinity of each farm. All samples were collected during the winter season (December to February) to reflect the actual operational temperatures of livestock facilities during cold months, as shown in Table 1. Immediately after collection, samples were placed in sterile containers, transported to the laboratory on ice within 6 h, and stored at 4 °C until further processing within 24 h.

### 2.2. Culture Medium Formulations

All medium components were weighed using an electronic balance (METTLER TOLEDO, Greifensee, Switzerland) and prepared using ultrapure water generated by an ultrapure water system (Millipore, Bedford, MA, USA). The pH values were measured using a pH meter (Shanghai INESA Scientific Instrument Co., Ltd., Shanghai, China). Unless otherwise specified, the prepared media were sterilized using a high-pressure steam sterilizer (Sanyo Electric Co., Ltd., Osaka, Japan).

Beef extract-peptone (NB) medium and De Man, Rogosa, and Sharpe (MRS) medium were used for bacterial isolation, Gauze’s No. 1 synthetic medium for actinomycete isolation, and potato dextrose broth (PDB) medium for fungal isolation. H_2_S-selective medium and NH_3_-selective medium were used for the screening of functional deodorizing strains.

Beef extract–peptone medium: 10 g beef extract and 5 g peptone were supplemented with 5 g NaCl. The mixture was diluted with distilled water to a final volume of 1000 mL.

MRS medium: The medium contained 0.04 g MnSO_4_, 8 g beef extract, 2 g K_2_HPO_4_, 0.2 g triammonium citrate, 20.0 g glucose, 5 g sodium acetate, 4 g yeast extract powder, 1 mL Tween 80, 10.0 g peptone, and 0.2 g MgSO_4_. The pH value was adjusted to 6.1–6.3, and the solution was brought to 1000 mL with distilled water.

Gauze’s No. 1 synthetic medium: The medium consisted of 20 g soluble starch, 1 g KNO_3_, 0.5 g K_2_HPO_4_, 0.5 g MgSO_4_·7H_2_O, 0.5 g NaCl, and 0.01 g FeSO_4_·7H_2_O. The pH was adjusted to 7.2–7.4, and the final volume was fixed to 1000 mL using distilled water.

PDB medium: 4 g potato extract powder, 20 g glucose, and 10 g peptone were dissolved in distilled water, and the volume was adjusted to 1000 mL.

H_2_S-selective medium: 2.0 g ammonium chloride, 0.5 g magnesium chloride, 3.0 g diammonium hydrogen phosphate, and 0.2 g calcium chloride were dissolved in distilled water to a final volume of 1000 mL. After autoclave sterilization, 10.0 g of sodium sulfide, completely dissolved in distilled water, was added aseptically through a membrane filter.

NH_3_-selective medium: 50 g sucrose, 2 g dipotassium hydrogen phosphate, 0.5 g magnesium sulfate, 0.1 g ferrous sulfate, and 0.5 g sodium chloride were dissolved in 600 mL of distilled water. Subsequently, 5 mL of 1% zinc sulfate solution was added, and the mixture was diluted to 1000 mL with distilled water. After autoclave sterilization, 10 mL of NH_3_ solution was added aseptically via membrane filtration.

### 2.3. Temperature Selection Rationale

Based on the winter sampling temperatures recorded in Table 1 (ranging from 5 °C to 15 °C), all subsequent isolation and functional assays were conducted at 15 °C. This temperature represents the upper boundary of the in situ winter temperature range and was identified as the minimum temperature at which the isolates retained detectable metabolic activity in preliminary tests.

### 2.4. Strain Isolation

The sample pretreatment procedure was performed as follows: 10 g of each sample was mixed with 90 mL of sterile physiological saline in a conical flask. The mixture was incubated in a shaking incubator (Sanyo Electric Co., Ltd., Osaka, Japan) at 180 r/min for 2 h to fully elute microorganisms and form a homogeneous bacterial suspension. The obtained suspension was serially diluted 10-fold with sterile physiological saline to prepare gradient dilutions ranging from 10^−1^ to 10^−9^. Next, 100 μL of each dilution was uniformly spread onto the surfaces of Gauze’s No. 1 synthetic medium, NB medium, PDB medium, and MRS medium using sterile spreaders (Eppendorf SE, Hamburg, Germany; Thermo Fisher Scientific, Waltham, MA, USA). Two biological replicates were set for each medium to ensure experimental accuracy. All inoculated plates were incubated at a constant temperature of 15 °C for three days. This temperature was chosen based on the in situ sampling temperatures recorded during winter (Table 1), which ranged from 5 °C to 15 °C, with 15 °C representing the upper boundary of the winter operational temperature spectrum for in-house facilities. Well-grown single colonies with distinct morphological characteristics and colors were selected and purified. A three-zone streaking method was applied for continuous purification for five rounds on the corresponding media under the same incubation conditions to obtain pure single-strain cultures.

### 2.5. Preliminary Screening of Deodorizing Functional Strains

The purified strains were inoculated into liquid medium and incubated at a constant temperature of 15 °C with shaking at 180 rpm for 3 days (Thermo Fisher Scientific, Waltham, MA, USA) to prepare stable bacterial suspensions.

Briefly, 10 mL of NH_3_-selective medium was transferred into a shake flask, followed by the addition of 10 μL of 25% NH_3_ solution pre-filtered using a 0.22 μm filter membrane. Afterward, 500 μL of the bacterial suspension was inoculated into the shake flask. The flask was sealed and incubated continuously on a shaker at 15 °C and 200 rpm for 5 days. When the incubation period finished, the appearance of turbidity in the culture broth demonstrated the NH_3_-degrading capacity of the strain and vice versa.

Then, 100 μL of the culture broth was evenly spread onto the surface of solid H_2_S-selective medium with a disposable spreader. The agar plates were incubated upside down at 15 °C for 3 days prior to observation. Strains showing robust growth on the H_2_S-selective medium were regarded as having the potential to degrade and utilize H_2_S.

The primary high-throughput screening served only to preliminarily exclude inactive strains. Positive candidates were then quantitatively re-tested to confirm genuine deodorizing capacity by directly measuring residual NH_3_ and H_2_S.

### 2.6. Secondary Screening of Deodorizing Functional Strains

The dominant strains obtained from preliminary screening were activated. The activated strains were inoculated into 200 g of fresh feces at 10% (*w*/*w*). The inoculated feces were placed into a 2 L sealed plastic barrel with a 50 mL sterile centrifuge tube containing 20 mL of 2.0% boric acid solution inside to absorb NH_3_ released from the feces. Then the barrels were incubated in a constant-temperature fermentation chamber at 15 °C, compared to a control group with an equal volume of liquid medium. Each treatment was performed in triplicate.

During incubation, the NH_3_ release of all groups was measured every 5 days using the boric acid absorption Kjeldahl method [15]. Finally, the NH_3_ removal rate was calculated from the measurements. The formula is as follows:

(1) $$\eta_{{NH}_{3}}=\frac{\left(C_{blank}-C_{treat}\right)}{C_{blank}}\times 100\%$$

where $\eta_{{NH}_{3}}$ stands for NH_3_ removal rate, %; $C_{blank}$ is the NH_3_ emission of the blank group, mg/m^3^; and $C_{treat}$ is the NH_3_ emission of the treatment group, mg/m^3^.

The pre-screened dominant strains were activated in liquid medium and then inoculated into 200 g of fresh feces at 10% (*w*/*w*). The mixture was transferred into a 2 L lidded plastic barrel. A 50 mL sterile centrifuge tube and a small beaker were placed inside each barrel, and the beaker was filled with 20 mL of zinc–amine complex absorbent to capture H_2_ S. The barrels were sealed and incubated at 15 °C in a constant-temperature chamber.

A control group supplemented with an equal volume of liquid medium was set up, and each treatment was performed in triplicate. The H_2_S emission of all groups was measured every 5 d via the zinc–amine complex absorption colorimetry [16]. The H_2_S removal rate was then calculated. The formula is as follows:

(2) $$\eta_{H_{2}S}=\frac{\left(C_{blank^{\prime }}-C_{{treat}^{\prime }}\right)}{C_{blank}}\times 100\%$$

where $\eta_{H_{2}S}$ stands for H_2_S removal rate, %; $C_{blank^{\prime }}$ is the H_2_S emission of the blank group, mg/m^3^; $C_{{treat}^{\prime }}$ is the H_2_S emission of the treatment group, mg/m^3^. Comparison of NH_3_ and H_2_S removal rates enabled the selection of high-efficiency deodorizing strains for developing compound bacterial preparations.

Determination of NH_3_: The boric acid absorption Kjeldahl method was used to measure NH_3_ emission. Firstly, 20 mL of 2.0% boric acid solution was used to absorb NH_3_ released from the treatment groups. Then, 50 μL of methyl red-bromocresol green was added as the indicator. The solution was titrated with standard hydrochloric acid solution until the color changed from blue-green to pale red, and the consumption of hydrochloric acid solution was recorded. The formula is as follows:

(3) $$C=\frac{\left(c\times v\times 2\times 1000\times 17\right)}{V}$$

where C stands for NH_3_ concentration, mg/L; c is the molar concentration of acid solution, mol/L; v is the volume of consumed acid solution, mL; 17 is the molar mass of NH_3_, g/mol; and V is the volume of 2% boric acid absorbent, mL.

Determination of H_2_S: The zinc–amine complex absorption colorimetry was adopted to determine H_2_S emission. A mixed chromogenic reagent was added after H_2_S was absorbed by the zinc–amine complex solution. The mixture was shaken well and kept for 30 min. Next, 2 drops of 40% diammonium hydrogen phosphate solution were added, and the absorbance was measured at a wavelength of 665 nm. The formula is as follows:

(4) $$C=\frac{(A-A_{0})Bs}{Vs}$$

where C stands for H_2_S concentration, mg/m^3^; A is the absorbance of the sample chromogenic solution; A_0_ is the absorbance of the blank solution; Bs is the reciprocal of the slope, μg/absorbance; and Vs is the sampling volume converted to standard state, L.

### 2.7. Antagonistic Assay and Strain Identification

Briefly, 200 μL of bacterial suspension was evenly spread onto NB agar plates. Sterilized filter paper discs were saturated with the test bacterial suspension and placed on the inoculated plates; blank control discs soaked in sterile water were set in parallel. The plates were incubated at 15 °C for 1–2 days. Then, antagonistic activity was examined, and strains exhibiting antagonism were excluded, while non-antagonistic strains were selected for the preparation of composite bacterial agents.

The 16S rRNA gene was amplified via PCR using the primer pair 27F (5′-AGAGTTTGATCCTGGCTCAG-3′) and 1492R (5′-GGTTACCTTGTTACGACTT-3′). The 20 μL PCR reaction system contained 2 μL of bacterial suspension, 1.0 μL of 27F primer (25 μmol/L), 1.0 μL of 1492R primer (25 μmol/L), 10.0 μL of 2×Taq PCR StarMix, and 6 μL of ddH_2_O.

The PCR cycling conditions were set as follows: initial denaturation at 95 °C for 3 min; 30 cycles of denaturation at 95 °C for 30 s, annealing at 55 °C for 30 s, and extension at 72 °C for 90 s; and a final extension at 72 °C for 5 min. The PCR products were sent to Youkang Biotechnology Co., Ltd. (Urumqi, Xinjiang, China) for sequencing. The obtained sequences were aligned with the NCBI GenBank database via the BLAST (version 2.17.0+) program to identify the most closely related strains, and a phylogenetic tree was constructed subsequently.

### 2.8. Preparation of Composite Deodorizing Agents and Practical Application Assays

The screened strains were cultured to logarithmic phase (approximately 10^8^–10^9^ CFU/mL) and mixed at equal volume ratios (1:1:1:1 for four-strain combinations and 1:1:1:1:1:1 for six-strain combinations) according to the formulation schemes shown in Table 2. The inoculated feces samples, with each strain mixture added at 10% (*w*/*w*) to 200 g of fresh feces, were transferred into a 2 L lidded plastic barrel. A commercial deodorizing agent (designated as Group S, consisting of Enterococcus faecalis and Bacillus subtilis; Haowangnong Biotechnology Co., Ltd., Henan, China) was used as a positive control. A 50 mL sterile centrifuge tube containing 20 mL of absorbent solution was placed inside each barrel. The barrels were tightly sealed and incubated at 15 °C in a constant-temperature chamber. Each treatment was performed in triplicate. After 10 days of incubation, gas emissions were measured, and the removal rates were calculated.

**Table 2:** Strain configuration of composite bacterial preparations.

Inoculant ID	NAC4 ^1^	GAA4	NCA7	NAC2	NBB8	NCC7
A	—	+	—	—	+	—
B	+	+	—	—	+	—
C	—	+	+	—	+	—
D	+	+	+	—	+	—
E	+	+	—	+	—	+
F	+	+	+	+	+	—
G	+	+	+	—	+	+
H	+	+	+	+	+	+
CK	—	—	—	—	—	—

^1^ “+” indicates the strain was included in the formulation; “—” indicates the strain was not included. CK, blank control group without bacterial inoculation.

The optimal composite agent identified from the above screening was further evaluated in two preliminary field application scenarios. For wastewater treatment, livestock wastewater (1000 mL) was dispensed into transparent 2 L plastic containers, and the composite agent was added at 5% and 10% (*v*/*v*) inoculation ratios, with an equal volume of sterile saline added to the control group. Each treatment was performed in triplicate. The containers were incubated under ambient low-temperature conditions (4–8 °C), and the physical state and odor of the wastewater were observed daily for 10 days. For farm spraying trials, a livestock house was divided into a treatment area and a control area with comparable conditions. The composite agent was sprayed in the treatment area, while the control area received no application. Odor gas concentrations (NH_3_ and H_2_S) were measured daily for 10 days post-application using a portable gas analyzer (Shandong Duorui Electronic Technology Co., Ltd., Shandong, China).

### 2.9. Data Analysis

Data processing and statistical analysis were performed using Microsoft Excel (Microsoft Corp., Redmond, WA, USA). All experiments were conducted with three biological replicates, and results are expressed as means ± standard deviation (SD). Statistical graphs were plotted with GraphPad Prism 9 (GraphPad Software, San Diego, CA, USA). One-way analysis of variance (ANOVA) followed by Dunnett’s multiple-comparison test was used to compare each treatment group with the blank control (CK). Degradation rates were calculated based on the difference between each treatment group and the CK control (Equations (1) and (2)). Statistical significance was defined at *p* < 0.05, with significance levels indicated as ns (*p* > 0.05); * (*p* < 0.05); ** (*p* < 0.01); *** (*p* < 0.001). Phylogenetic trees were constructed using MEGA 11.0 (MEGA Software, Tempe, AZ, USA).

## 3. Results

### 3.1. Strain Isolation Results

We isolated a total of 206 pure strains from the samples. NB medium yielded the highest number of strains (115), followed by Gauze’s No. 1 synthetic medium (55), PDB medium (31), and MRS medium (5). The low number of strains from the MRS medium may be attributed to the limited abundance of lactic acid bacteria in these samples. The detailed strain IDs for each sample and medium are presented in Table 3.

### 3.2. Preliminary Screening of Strains

The isolated strains were subjected to preliminary qualitative screening using NH_3_-selective and H_2_S-selective media. The results are shown in Table A1. Twenty-nine strains induced turbidity in NH_3_-selective medium, indicating their ability to utilize NH_3_. Twenty-six strains grew on H_2_S-selective medium supplemented with NA2S, demonstrating their capacity to degrade H_2_S. Among them, 10 strains exhibited both capabilities, being able to induce turbidity in NH_3_-selective medium and grow on NA2S-containing H_2_S-selective medium. However, we considered these primary screening results provisional, as growth on selective media may reflect tolerance rather than true degradation. We therefore performed quantitative confirmation of actual substrate removal through secondary screening.

### 3.3. Secondary Screening of Strains

**Figure 1:**
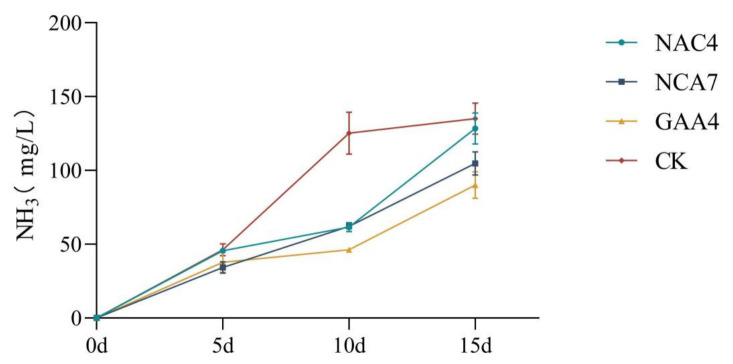
Ammonia release from the treatment group after the addition of the strain.

Strains positive in the preliminary screening were subjected to secondary screening. The results confirmed that not all primary-positive strains possessed genuine degradation activity. For example, strain NAC4 exhibited only 0.15% NH_3_ removal at day 5 (Table 4) despite showing positive turbidity in the primary qualitative test, indicating that growth-based primary screening alone would have produced false positives. Conversely, strains such as GAA4 showed substantial removal (14.65% at day 5 and 63.81% at day 10), confirming the validity of the quantitative secondary screening approach. Three high-efficiency-degrading strains were obtained and designated as NAC4, NCA7, and GAA4 (Figure 1). NH_3_ emissions were measured every 5 days (Table 4). On day 5, strain NCA7 showed the highest removal rate (24.87%). On days 10 and 15, strain GAA4 exhibited the highest efficiency, with removal rates of 63.81% and 41.43%, respectively. All three strains achieved their maximum degradation rates on day 10, indicating that the optimal degradation period was days 5–10.

**Figure 2:**
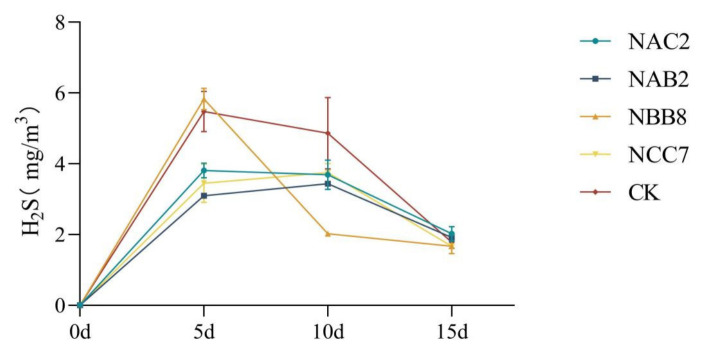
Hydrogen sulfide release from treated groups after the addition of bacterial strains.

Strains positive in the preliminary H_2_S screening were further screened. Four high-efficiency H_2_S-degrading strains were obtained and designated as NAB2, NAC2, NBB8, and NCC7 (Figure 2). H_2_S emissions were measured every 5 days (Table 5). On day 5, strain NAB2 had the highest removal rate, 43.51%, while strain NBB8 reached the maximum efficiency (58.38%) on day 10.

### 3.4. Antagonism Assay Results

**Figure 3:**
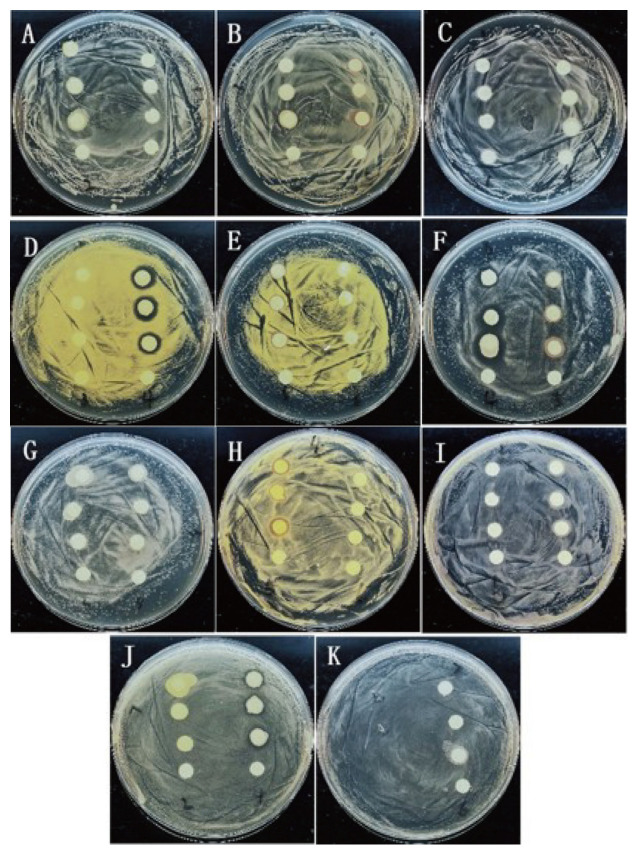
Antagonistic results between strains: (**A**) NAC4 vs. GAA4 (**left**) and NAC4 vs. NCA7 (**right**); (**B**) NAC4 vs. NAB2 (**left**) and NAC4 vs. NAC2 (**right**); (**C**) NAC4 vs. NBB8 (**left**) and NAC4 vs. NCC7 (**right**); (**D**) GAA4 vs. NCA7 (**left**) and GAA4 vs. NAB2 (**right**); (**E**) GAA4 vs. NAC2 (**left**) and GAA4 vs. NBB8 (**right**); (**F**) NCA7 vs. NAB2 (**left**) and NCA7 vs. NAC2 (**right**); (**G**) NCA7 vs. NBB8 (**left**) and NCA7 vs. NCC7 (**right**); (**H**) NAB2 vs. NAC2 (**left**) and NAB2 vs. NBB8 (**right**); (**I**) NAC2 vs. NBB8 (**left**) and NAC2 vs. NCC7 (**right**); (**J**) NCC7 vs. GAA4 (**left**) and NCC7 vs. NAB2 (**right**); (**K**) NCC7 vs. NBB8. Antagonistic zones are indicated by clear inhibition areas between colonies.

In the plate confrontation assay, obvious antagonism was observed between GAA4 and NAB2 (right of Figure 3D), NCA7 and NAB2 (left of Figure 3F), and NCC7 and NAB2 (right of Figure 3J). These strains grew normally when co-cultured but formed distinct antagonistic zones, indicating mutual repulsion. No significant antagonism was detected among the remaining strains. Their colonies grew normally with intermingled growth areas, and no clear antagonistic lines or inhibition zones were observed, showing no growth inhibition or repulsion.

### 3.5. Growth Characteristics of Selected Strains at 15 °C

**Figure 4:**
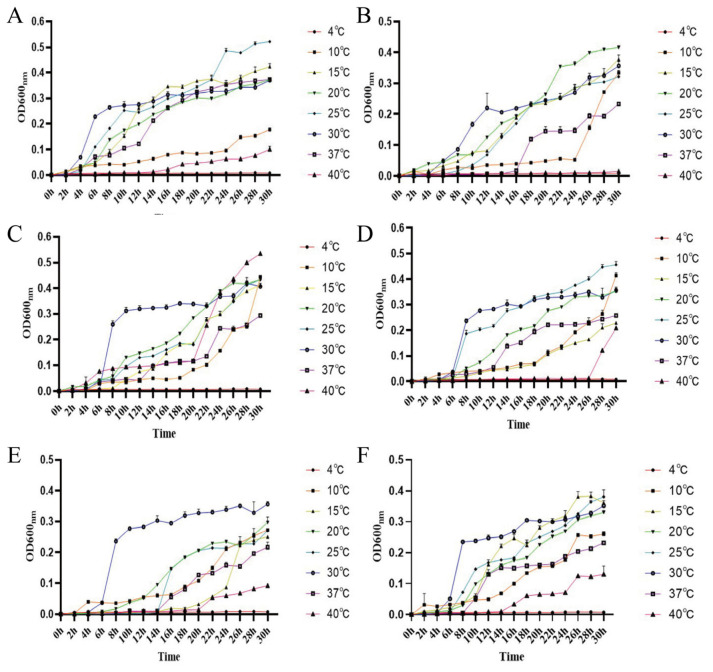
Growth curves of the six selected strains at different temperatures (4, 10, 15, 20, 25, 30, 37, and 40 °C): (**A**) NAC4; (**B**) GAA4; (**C**) NCA7; (**D**) NAC2; (**E**) NBB8; (**F**) NCC7. OD_600_ was measured at 2 h intervals over a 30 h period at each temperature. Each curve represents the mean of three replicate measurements.

To characterize the growth kinetics of the six selected strains at the experimental temperature (15 °C), each strain was cultured in liquid medium, and OD_600_ was measured at regular intervals over a 30 h period. The growth curves of the six strains at 15 °C are shown in Figure 4.

All six strains exhibited detectable growth at 15 °C, with distinct strain-specific growth patterns. NAC4 entered the exponential phase at 6 h and reached the stationary phase at 16 h. GAA4 showed a prolonged lag phase (0–12 h), followed by exponential growth from 12 to 18 h. NCA7 initiated growth at 8 h and exhibited a sustained linear increase through 24 h without a clear exponential burst. NAC2 showed a lag phase of 6 h followed by continuous growth through 24 h. NBB8 exhibited the most extended lag phase (0–18 h), followed by exponential growth from 18 to 26 h. NCC7 showed the shortest lag phase (0–4 h) and entered the stationary phase at 8 h.

### 3.6. Strain Identification Results

**Figure 5:**
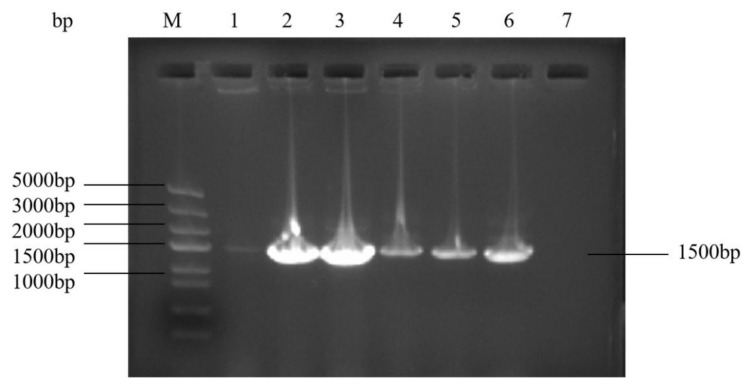
PCR amplification of 16S rDNA sequences of 6 strains of bacteria. Note: M, DNA standard DL5000; 1 to 6, NAC4, GAA4, NCA7, NAC2, NBB8, and NCC7 16S rDNA gene fragments; 7, negative control.

Using the bacterial suspensions of NAC4, GAA4, NCA7, NAC2, NBB8, and NCC7 as templates, the universal bacterial primers were applied for PCR amplification of 16S rDNA fragments. The products were detected by 1% agarose gel electrophoresis. The amplified fragments of all six strains were approximately 1500 bp in length, matching the expected size. The results are shown in Figure 5.

**Figure A1:**
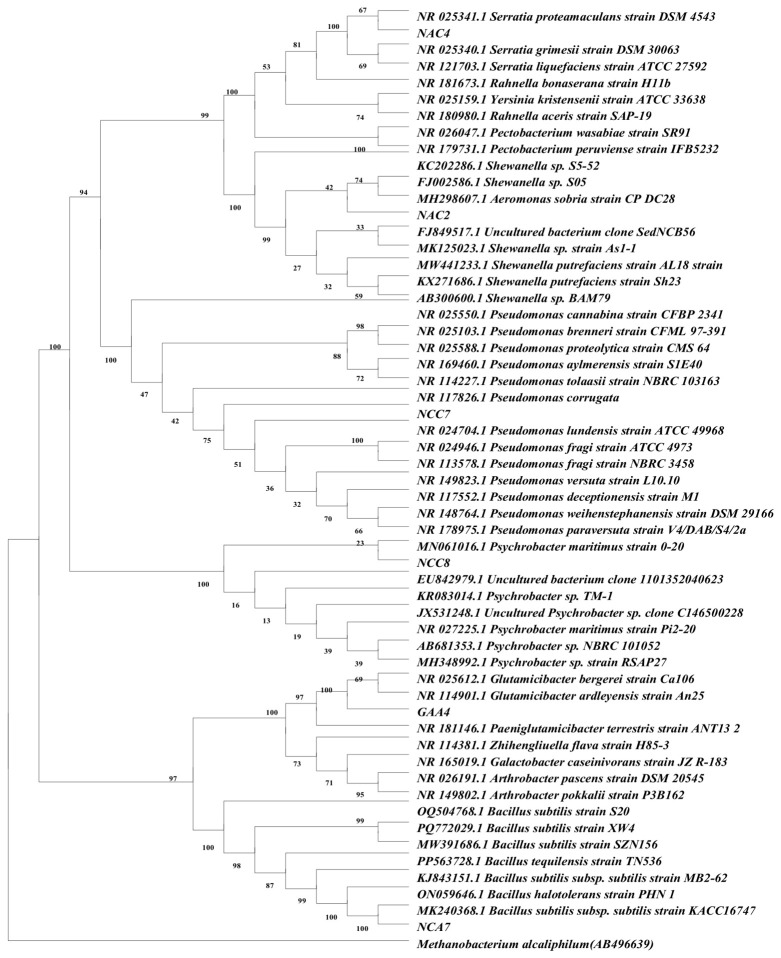
Phylogenetic tree based on 16S rDNA gene sequences showing the phylogenetic relationships of strains NAC4, GAA4, NCA7, NAC2, NBB8, and NCC7 with closely related type strains. The tree was constructed using the neighbor-joining method with p-distance as the nucleotide substitution model in MEGA 11.0. Bootstrap values (based on 1000 replicates) are shown at branch nodes. *Psychrobacter maritimus* was used as the outgroup. Bar, 0.02 substitutions per nucleotide position.

A phylogenetic tree based on the acquired 16S rDNA sequences was constructed (Figure A1), revealing that the high-efficiency deodorizing strains isolated from livestock feces in this study were identified as *Serratia proteamaculans*, *Glutamicibacter bergerei*, *Bacillus subtilis*, *Shewanella putrefaciens*, *Psychrobacter maritimus*, and *Pseudomonas* sp.

### 3.7. Performance of Mixed Bacterial Inoculants

Six screened strains were mixed in different combinations to prepare compound bacterial agents for further screening.

**Figure A2:**
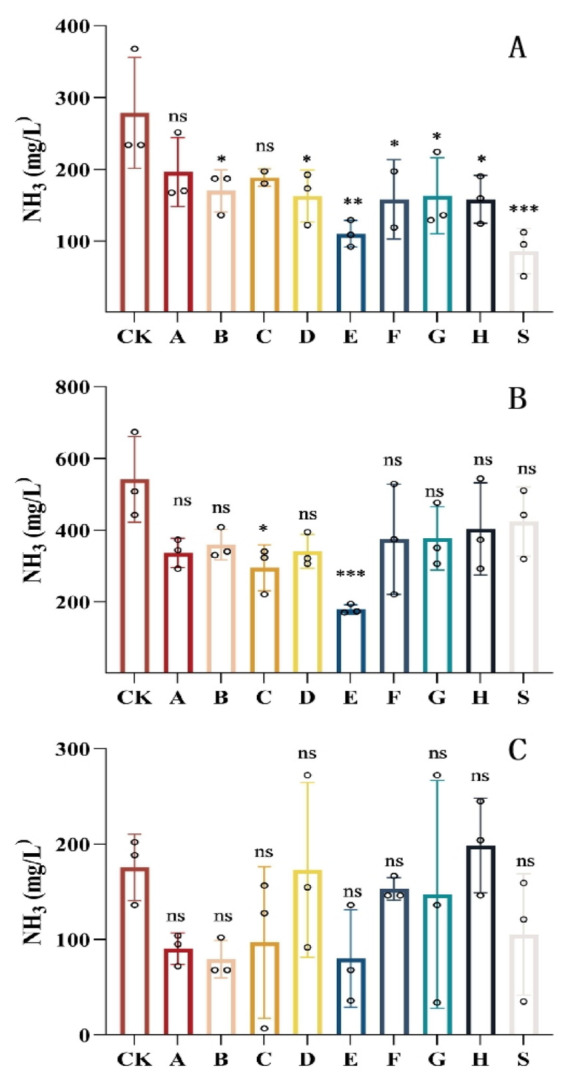
NH3 content was measured in the absorbent solution after treatment with the composite bacterial preparation: (**A**) measurements at 5 d of fungicide treatment; (**B**) measurements after 10 d of fungicide treatment; (**C**) measurements at 15 d after fungicide treatment. * (*p* < 0.05); ** (*p* < 0.01); *** (*p* < 0.001).

Eight compound bacterial agents were formulated using the 6 strains for screening. NH_3_ concentrations in the absorbing solution after treatment are presented in Table 6. The NH_3_ removal effect varied significantly among agents at days 5, 10, and 15. At day 5, NH_3_ concentrations of all agent groups were significantly lower than those of the control group (CK, 234–368 mg/L). Group E had the lowest concentration (92.4–129.2 mg/L) and showed excellent NH_3_ removal performance. At day 10, NH_3_ concentrations generally increased in all groups. However, Groups B and C still kept relatively low levels (136–197.2 mg/L), reflecting good stability. At day 15, concentrations in Groups C and E dropped significantly (Group C: 6.8–156.4 mg/L; Group E: 36–136 mg/L), indicating their stronger capacity for NH_3_ removal in long-term treatment. Overall, the compound bacterial agents C and E performed well in NH_3_ removal across all treatment phases, demonstrating distinctly higher efficacy than other groups during the long-term treatment period (day 15). With excellent NH_3_ removal capacity, Groups C and E are ideal candidate agents for practical application (Figure A2).

**Figure A3:**
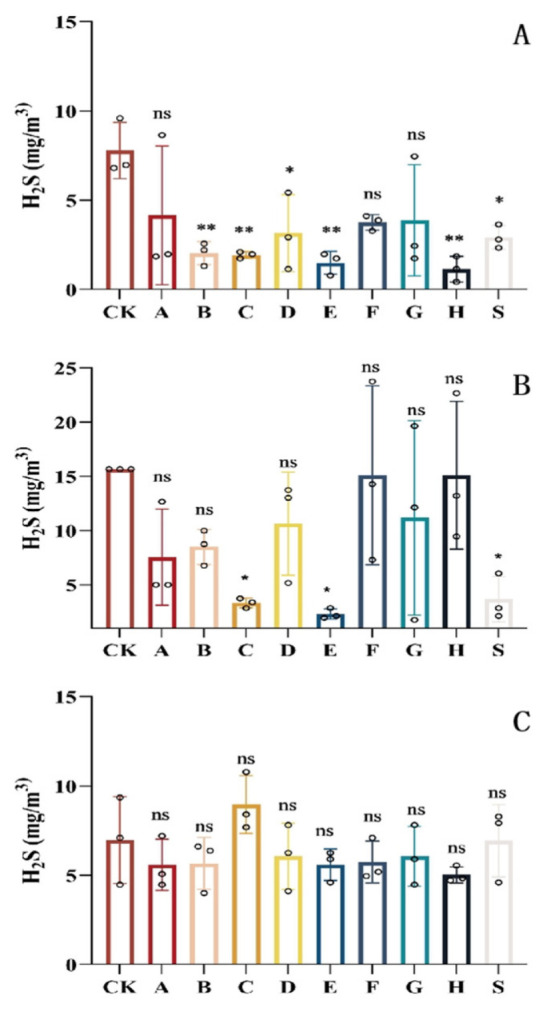
Content of H_2_S measured in the absorbent solution after treatment with the composite bacterial preparation: (**A**) measurements at 5 d of fungicide treatment; (**B**) measurements after 10 d of fungicide treatment; (**C**) measurements at 15 d after fungicide treatment. * (*p* < 0.05); ** (*p* < 0.01).

For H_2_S removal, H_2_S concentrations in the absorbing solution after treatment are presented in Table 7. At day 5, all compound bacterial agent groups showed significantly lower H_2_S concentrations than the control group (CK, 6.8–9.6 mg/m^3^), with Groups E and H showing the lowest concentrations (Group E: 0.8–2.0 mg/m^3^; Group H: 0.4–1.9 mg/m^3^). At day 10, H_2_S concentrations generally increased across groups, but Groups C and E remained at relatively low levels (Group C: 2.9–3.8 mg/m^3^; Group E: 2.0–2.9 mg/m^3^). At day 15, Groups E and H maintained relatively low H_2_S concentrations (Group E: 4.6–6.3 mg/m^3^; Group H: 4.7–5.5 mg/m^3^). Overall, Groups E and H demonstrated superior H_2_S removal performance across all treatment phases, particularly at the early stage (day 5) (Figure A3).

The degradation rates of NH_3_ and H_2_S for all treatment groups are summarized in Table 8. At day 5, Groups E and H showed relatively high degradation rates for both NH_3_ and H_2_S (Group E: NH_3_ 60.48%, H_2_S 75.89%; Group H: NH_3_ 58.11%, H_2_S 81.63%). At day 10, Group E achieved the highest degradation rates for both NH_3_ (66.92%) and H_2_S (85.25%), significantly outperforming other groups. At day 15, Group E maintained a relatively high NH_3_ degradation rate (54.41%), while its H_2_S degradation rate declined to 19.95%. Based on these results, Agent E was selected as the optimal composite formulation for further evaluation.

To preliminarily evaluate the practical applicability of the optimal composite agent (designated Agent E), it was applied in two field scenarios: livestock wastewater treatment and farm spraying.

**Figure 6:**
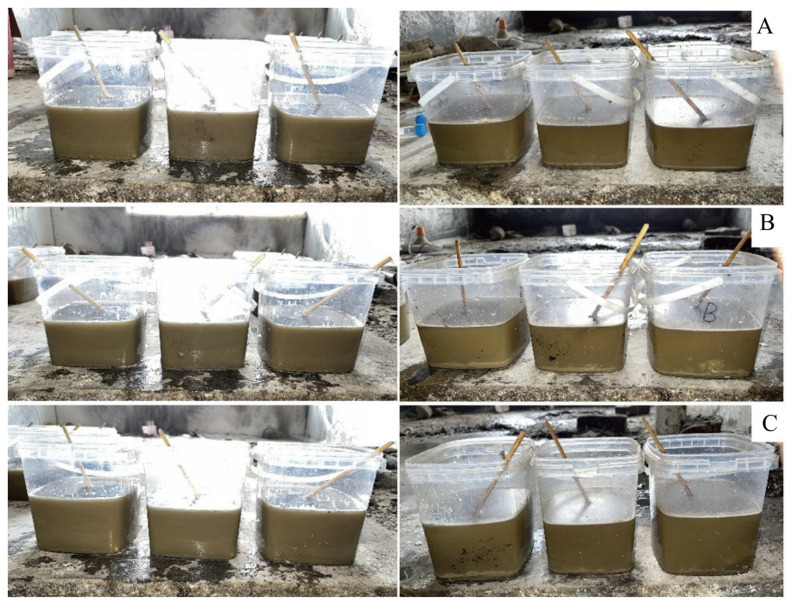
Effect of Agent E on livestock wastewater: (**A**) control; (**B**) 5% inoculum; (**C**) 10% inoculum. Left panel: day 0; right panel: day 10.

For wastewater treatment, Agent E was added to livestock wastewater at 5% and 10% (*v*/*v*) inoculation ratios, with an equal volume of sterile saline added to the control group. After 10 days of incubation at ambient low temperature (4–8 °C), the 10% inoculum treatment showed visible improvement in clarity and reduction in odor compared to the control and 5% treatment groups, while no obvious difference was observed between the 5% treatment and the control (Figure 6).

For farm spraying, Agent E was sprayed in a livestock house, with an adjacent untreated area of comparable conditions serving as the control. Odor gas concentrations were monitored using a portable gas analyzer over 10 days post-application. H_2_S concentrations remained below the detection limit (0 μmol/mol) in both the treated and control areas throughout the trial period, indicating that NH_3_ was the predominant odorant in this farming environment. As shown in Table 9, NH_3_ concentrations in the treated area were comparable to the control for the first 2 days and then decreased below control levels from day 3 through day 7 (6 vs. 8–9 μmol/mol). From day 8 onward, concentrations in both areas declined to approximately 3 μmol/mol, attributed to a sudden drop in ambient temperature to 0 °C, which suppressed microbial activity in both the agent and the indigenous microbial community.

## 4. Discussion

In recent years, the scale and intensity of China’s livestock and poultry farming industry have increased steadily, leading to a steady rise in total manure emissions and increasingly severe environmental pollution from farming waste. Statistical data show that the total output of livestock and poultry manure in China has been growing steadily since 2011, exceeding 1.0 billion tons by 2020. Over this decade, the increase reached 36.35 million tons, representing an overall growth rate of 57.1% [17]. Emissions from cattle, sheep, pigs, and poultry have all shown significant upward trends, with particularly prominent growth rates for poultry and swine, at 96.1% and 96.6%, respectively [18]. Untreated livestock manure continuously releases typical odorous gases such as ammonia and hydrogen sulfide. These emissions not only degrade regional air quality and cause odor-related public complaints but also threaten the respiratory and overall health of both humans and animals through aerosol diffusion and substance migration, making them a core environmental issue urgently requiring resolution in intensive farming areas [19].

Compared with physical and chemical deodorization methods, microbial deodorization has become a mainstream technology for controlling odors from livestock manure due to its core advantages of environmental friendliness, strong sustainability, and low cost. The fundamental principle is that microorganisms convert nitrogen- and sulfur-containing odorous substrates into inorganic, harmless products through their metabolic activities [20]. However, most existing deodorizing microbial resources are mesophilic (normal-temperature) strains. Under low-temperature conditions, their membrane fluidity decreases, key metabolic enzymes are inhibited, and metabolic pathways are disrupted, leading to a substantial loss of deodorization function [21].

In this study, conducted in the cold-region farming environment of Xinjiang (elevation approximately 300–500 m), five low-temperature deodorizing bacterial strains capable of efficiently degrading ammonia and hydrogen sulfide were successfully screened. Deodorization performance tests on individual strains showed that the ammonia degradation rates ranged from 50.80% to 63.81%, and the hydrogen sulfide degradation rates ranged from 43.52% to 57.50%. These cold-tolerant strains employ mechanisms such as synthesizing cold-adaptive enzymes, regulating cell membrane fatty acid composition, and accumulating compatible solutes to withstand low-temperature stress and maintain stable ammonification and sulfide oxidation metabolic pathways, thereby ensuring continuous degradation of odorous substrates in cold environments [22]. Compared with existing studies on mesophilic deodorizing strains, the low-temperature adaptability of the strains screened in this study is notably advantageous. In contrast, the strains from this study demonstrate superior ammonia removal performance at low temperatures, while their hydrogen sulfide degradation capacity is comparable to that of efficient mesophilic strains. Although the mesophilic strains screened by Li et al. achieved removal rates for odorous gases exceeding 90%, offering superior ultimate deodorization efficiency, these strains are only suitable for normal-temperature farming environments [23]. Under the prolonged low-temperature stress typical of Xinjiang winters, they would likely suffer from activity decay and metabolic stagnation, making them unsuitable for practical application in cold regions.

Single microbial strains often suffer from limitations such as narrow metabolic functions, weak environmental resilience, and poor deodorization stability, making it difficult to achieve sustained and efficient odor removal [24]. In contrast, a bacterial consortium prepared by strain compounding can enhance deodorization performance through functional complementarity and synergistic coupling of metabolic pathways. Different strains can target nitrogen- and sulfur-containing odorous substrates, respectively. At the same time, their symbiotic interactions can further elevate the overall expression level of low-temperature enzyme systems, alleviating the limitations of single-strain metabolism under cold conditions [25]. In this study, the screened low-temperature functional strains were optimized into a consortium to prepare a composite deodorizing agent, designated Agent E. Under low-temperature conditions, this agent achieved ammonia and hydrogen sulfide degradation rates of 52.67% and 78.74%, respectively. Compared with the single strains, the hydrogen sulfide degradation efficiency was significantly enhanced, and overall deodorization stability and comprehensive performance were markedly improved, confirming that strain compounding is an effective strategy for enhancing low-temperature deodorization. This result is consistent with the findings of Wang et al., who prepared a composite microbial agent (MIX) using multiple strains that also effectively degraded ammonia and hydrogen sulfide, supporting the concept that multi-strain synergy can enhance the deodorization capacity of manure odors [26]. Furthermore, some studies have shown that an equal-proportion compounding system of multiple strains can maximize synergistic deodorization, providing a strong theoretical basis for the compounding approach used in this study [24].

The deodorization efficiency of a microbial system is not constant; it varies dynamically with factors such as incubation period, environmental conditions, and application method. This dynamic is intrinsically linked to microbial metabolic activity, enzymatic reaction rates, and substrate utilization patterns [27]. Our results indicate that the high-efficiency deodorization period for the composite agent is concentrated between 5 and 10 days. This temporal pattern correlates with the growth kinetics of the component strains at 15 °C, where their exponential phases occurred during the initial incubation stage, corresponding to the peak substrate consumption period. During this phase, the strains are in the logarithmic growth phase, exhibiting high low-temperature degradation enzyme activity and optimal catalytic decomposition efficiency for ammonia and hydrogen sulfide. The complementary growth patterns among strains—with some entering the exponential phase earlier (e.g., NCC7) and others sustaining growth longer (e.g., NAC2, NCA7)—likely contribute to the extended overall deodorization performance of the composite agent.

Therefore, day 10 was determined as the optimal time point for performance evaluation. The transition from laboratory-scale performance to practical field application warrants careful consideration. In this study, Agent E achieved NH_3_ and H_2_S removal rates of 66.92% and 85.25% at day 10 under controlled 15 °C conditions using fresh feces in sealed barrels. Encouragingly, preliminary field trials demonstrated that Agent E retains deodorizing activity under real farming conditions: in livestock wastewater, the 10% inoculum treatment showed visible clarity improvement and odor reduction after 10 days; in farm spraying trials, NH_3_ concentrations in the treatment area remained lower than in the control area from day 3 to day 7 post-application. However, field performance was less pronounced than laboratory results, primarily due to lower ambient temperatures (4–8 °C during the trial period), which approached the lower growth threshold of the component strains, as well as the open environment that allows gas dispersion and introduces competing indigenous microorganisms. This discrepancy between laboratory and field performance is commonly observed in microbial deodorant applications. It highlights the need for practical implementation strategies such as periodic reapplication every 5–7 days and application during periods when ambient temperatures exceed 10 °C. Despite these challenges, the field trials provide encouraging evidence that Agent E has practical potential for winter deodorization in livestock houses, where temperatures are typically moderated by animal body heat to approximately 10–15 °C.

Comparing our results with those from other studies, the deodorization efficiency of microbial agents tends to fluctuate and decline with prolonged incubation time. This is primarily attributed to the gradual consumption of available odorous substrates, imbalance in the microbial community, and feedback inhibition caused by the accumulation of metabolic byproducts, which collectively reduce enzymatic reaction rates. Considering practical engineering applications, a single one-time application of a microbial agent is insufficient to achieve long-term stable deodorization. Instead, an operational strategy involving a moderate increase in inoculum size and periodic reapplication every 5 to 10 days can sustainably supplement active functional populations and maintain a high level of metabolic activity, effectively compensating for the decline in microbial activity and further improving odor control in cold-region farming environments. Previous studies have confirmed that factors such as strain composition, inoculum size, application method, and ambient temperature are key regulators influencing microbial deodorization efficiency [27,28,29].

This study has several limitations. First, the sample size (*n* = 12 from 5 farms in three locations) and winter-only sampling limit the generalizability of our findings, and broader sampling across seasons and regions is needed for validation. Second, the deodorization performance was primarily evaluated under laboratory conditions. While preliminary field trials (wastewater treatment and farm spraying) showed positive results, long-term, large-scale validation is still needed. Additionally, we focused primarily on NH_3_ and H_2_S as representative odorants and did not systematically evaluate other odorants, including VOCs, mercaptans, and sulfides. The molecular mechanisms underlying cold resistance and synergistic metabolic networks within the consortium also remain unclear. Future work will address these gaps through expanded sampling, field-scale trials, and mechanistic studies, including genomic and transcriptomic analyses.

## 5. Conclusions

In this study, three bacterial strains (N24, G4, and N75) capable of degrading ammonia in manure at low temperatures were obtained, identified as *Serratia proteamaculans*, *Glutamicibacter halophytocola*, and *Shewanella putrefaciens*. Their degradation rates peaked on day 10 at 52.62%, 63.81%, and 50.80%, respectively. Two Pseudomonas strains (N16 and N56) capable of degrading hydrogen sulfide at low temperatures were also obtained. Strain N16 reached its highest degradation rate of 43.52% on day 5, while strain N56 reached 57.50% on day 10. A deodorizing microbial agent E was then prepared, showing an ammonia degradation rate of 52.67% and a hydrogen sulfide degradation rate of 78.74%. Thus, an effective combination of deodorizing agents was identified.

## Figures and Tables

**Table 1:** Information on collected samples and in situ sampling temperatures.

Sampling Site	Farm Type	Sample Type	Sample ID	Sampling Temperature
Shihezi	Cattle farm	manure	AA	13 °C
Shihezi	Cattle farm	manure	AB	12 °C
Shihezi	Cattle farm	compost	AC	35 °C
Shihezi	Cattle farm	soil	AD	6 °C
Shihezi	Cattle farm	wastewater	AE	13 °C
Shihezi	Cattle farm	compost	AF	7 °C
Shihezi	Cattle farm	wastewater	AG	13 °C
Shawan	Sheep farm	compost	BA	9 °C
Shawan	Sheep farm	wastewater	BB	15 °C
Shihezi	Pig farm	manure	CA	5 °C
Shihezi	Pig farm	wastewater	CB	5 °C
Shihezi	Poultry farm	sludge	CC	5 °C

**Table 3:** Isolation results of strains and ID.

Sample ID	NB ^1^	PDB	MRS	G1
AA	NAA1~NAA13	PAA1	-	GAA1~GAA4
AB	NAB1~NAB7	PAB1~PAB4	MAB1	GAB1~GAB6
AC	NAC1~NAC5	PAC1~PAC3	-	GAC1~GAC7
AD	NAD1~NAD4	-	MAD1~MAD2	GAD1~GAD8
AE	NAE1~NAE4	-	-	GAE1~GAE4
AF	NAF1~NAF8	-	-	GAF1~GAF3
AG	NAG1~NAG13	PAG1~PAG2	-	GAG1~GAG8
BA	NBA1~NBA6	PBA1~PBA3	-	GBA1~GBA5
BB	NBB1~NBB8	PBB1~PBB2	MBB1	GBB1~GBB5
CA	NCA1~NCA15	PCA1~PCA7	-	-
CB	NCB1~NCB8	PCB1~PCB4	MCB1	GCB1~GCB4
CC	NCC1~NCC24	PCC1~PCC5	-	GCC1

^1^ NB, nutrient broth medium; PDB, potato dextrose broth medium; MRS, de Man, Rogosa, and Sharpe medium; G1, Gauze’s No. 1 synthetic medium. “-” indicates no strains were isolated from this sample on the corresponding medium.

**Table 4:** Measurement results of the NH_3_ degradation rate.

Strain Number ^1^	Day 5	Day 10	Day 15
NAC4	0.15%	52.62%	14.29%
GAA4	14.65%	63.81%	41.43%
NCA7	24.87%	50.80%	28.57%

^1^ Data represent the means of three biological replicates. CK, blank control group without bacterial inoculation. All three strains showed significantly higher NH_3_ removal compared to the CK control at day 10 (*p* < 0.05, Dunnett’s test).

**Table 5:** Hydrogen sulfide degradation rate measurement results.

Strain Number ^1^	Day 5	Day 10	Day 15
NAB2	43.51%	29.35%	−7.35%
NAC2	30.48%	24.11%	−13.35%
NBB8	−6.39%	58.38%	6.65%
NCC7	37.00%	22.88%	6.65%

^1^ Data represent the means of three biological replicates. CK, blank control group without bacterial inoculation. Negative values indicate that H_2_S emission in the treatment group temporarily exceeded that of the CK control during the early or late incubation phase, prior to the establishment or after the decline in active degradation. All four strains showed significantly higher H_2_S removal compared to the CK control at day 10 (*p* < 0.05, Dunnett’s test).

**Table 6:** Treatment effect of compound bacterial preparation on NH_3_ gas.

Treatment	NH_3_ Concentration After 5 d (mg/L)	NH_3_ Concentration After 10 d (mg/L)	NH_3_ Concentration After 15 d (mg/L)
A	167.28~251.6	292.4~373.2	72~95.2
B	136~187	136~187	68~102
C	180.2~197.2	180.2~197.2	6.8~156.4
D	122.4~192.4	306~394.4	91.8~272
E	92.4~129.2	170~193.8	36~136
F	119.0~197.2	221~528.7	146.2~166.6
G	129.2~224.4	306~476	34~272
H	124.1~190.4	292.4~544	146.2~244.8
S ^1^	51~112.2	319.6~510	34.9~159.2
CK	234~368	442~674	136~202

^1^ S represents a commercial deodorizing agent (Haowangnong, containing Enterococcus faecalis and Bacillus subtilis). CK, blank control group without bacterial inoculation. The data represent different ranges from three biological replicates. All treatment groups showed significantly lower NH_3_ concentrations compared to the CK control (*p* < 0.05, Dunnett’s test).

**Table 7:** Treatment effect of compound bacterial preparation on H_2_S gas.

Treatment	H_2_S Concentration After 5 d (mg/L)	H_2_S Concentration After 10 d (mg/L)	H_2_S Concentration After 15 d (mg/L)
A	1.9~8.6	5.0~12.7	4.5~7.2
B	1.3~2.6	6.8~10.0	4.0~6.6
C	1.7~2.1	2.9~3.8	7.7~10.8
D	1.1~5.4	5.2~13.8	4.1~7.8
E	0.8~2.0	2.0~2.9	4.6~6.3
F	3.3~4.1	7.3~23.8	5.0~7.1
G	1.7~7.5	1.8~19.6	4.5~7.8
H	0.4~1.9	9.5~22.7	4.7~5.5
S ^1^	2.3~3.6	2.1~6.1	4.6~8.3
CK	6.8~9.6	15.7	4.5~9.4

^1^ S represents a commercial deodorizing agent (Haowangnong, containing Enterococcus faecalis and Bacillus subtilis). CK, blank control group without bacterial inoculation. The data represent different ranges from three biological replicates.

**Table 8:** Degradation rate of malodorous gas by composite bacterial preparation.

Treatment	NH_3_ (%)			H_2_S (%)		
	5 d	10 d	15 d	5 d	10 d	15 d
A	29.56	37.83	48.48	33.16	51.82	19.95
B	39.00	33.63	54.79	67.28	45.74	18.82
C	32.28	49.75	61.73	68.88	78.79	−28.37
D	41.60	37.09	1.50	49.11	32.07	13.13
E	60.48	66.92	54.41	75.89	85.25	19.95
F	43.27	30.75	10.87	39.54	31.12	13.70
G	41.44	30.28	16.03	37.63	28.65	13.13
H	58.11	48.50	14.74	81.63	3.58	27.91
S ^1^	69.09	21.70	40.09	52.93	76.51	0.62

^1^ S represents a commercial deodorizing agent (Haowangnong, containing Enterococcus faecalis and Bacillus subtilis). CK, blank control group without bacterial inoculation. The data represent different ranges from three biological replicates.

**Table 9:** NH_3_ concentrations (μmol/mol) in treated and control areas after spraying Agent E.

Day Post-Application ^1^	Treated Area	Control Area
1	6	6
2	8	8
3	6	8
4	6	8
5	6	9
6	6	8
7	6	8
8	3	3
9	3	3
10	5	6

^1^ NH_3_, ammonia; H_2_S, hydrogen sulfide (H_2_S remained below the detection limit of 0 μmol/mol in both areas throughout the trial period). The data represent single measurements from field trials. The treated area received Agent E spraying; the control area received no application.

## Data Availability

The datasets generated and analyzed during the current study are available from the corresponding author upon reasonable request.

## Appendix A

**Table A1:** Results of initial screening of strains.

Inoculant ID	NH_3_	H_2_S
GAA3	++	−
GAA4	++	+
GAB4	−	+
GAC7	+++	+
GAD4	++	−
GAE1	+++	−
GAE4	−	+
GAG8	−	+
GBA3	−	+
GBA4	+++	−
GBB2	−	+
MCB1	++	+
NAA2	+++	−
NAA3	++	−
NAA6	−	+
NAA7	++	−
NAA10	++	+
NAA11	+++	+
NAB2	++	+
NAB5	+++	−
NAB6	+++	−
NAC2	−	+
NAC4	+++	+
NAE3	−	+
NAF2	++	−
NAF6	++	−
NAF7	+	−
NAG3	++	+
NAG4	−	+
NAG12	+++	−
NBA2	−	+
NBA4	+++	−
NBB2	++	−
NBB8	−	+
NCA2	+++	−
NCA7	++	+
NCA9	−	+
NCA13	−	+
NCC5	++	−
NCC7	++	+
NCC9	−	+
NCC12	++	−
NCC15	−	+
NCC16	−	+
NCC20	−	+
PAB4	+++	−

“−” indicates no detectable deodorizing activity, whereas “+”, “++”, and “+++” indicate weak, moderate, and strong deodorizing activities against NH_3_ or H_2_S, respectively.

## References

[1] Khanal P., Dhakal R., Khanal T., Pandey D., Devkota N.R., Nielsen M.O. (2022). Sustainable Livestock Production in Nepal: A Focus on Animal Nutrition Strategies. Agriculture.

[2] Han D.X., Yan X.J., Ni J.Q., Zhao H.X., Wang K.Y. (2025). Evaluation of different strategies for swine house exhaust odor mitigation. Environ. Int..

[3] Minami K., Takahashi A., Sakurai K., Mikasa H., Takasaki M., Doshu N., Aoyama K., Nakamura T., Iwai R., Kawamoto T. (2022). Apparatus for ammonia removal in livestock farms based on copper hexacyanoferrate granules. Biosyst. Eng..

[4] Nie E.Q., Zheng G.D., Ma C. (2020). Characterization of odorous pollution and health risk assessment of volatile organic compound emissions in swine facilities. Atmos. Environ..

[5] Yan Z.Y., Li J., Liu X.F., Yuan Y.X., Liao Y.Z., Li X.D. (2017). Deodorization of swine manure using a *Lactobacillus* strain. Environ. Eng. Manag. J..

[6] Szwedziak K., Grzywacz Z., Wrotkowski K. (2018). The Application of Microbial Additive in Poultry Production as a Way to Reduce Emission of Harmful Gases into the Environment. Rocz. Ochr. Sr..

[7] Muñoz R., Malhautier L., Fanlo J.L., Quijano G. (2015). Biological technologies for the treatment of atmospheric pollutants. Int. J. Environ. Anal. Chem..

[8] Guan J.Y., Yao J.Q., Li M.Y., Zheng J.H. (2021). Assessing the Spatiotemporal Evolution of Anthropogenic Impacts on Remotely Sensed Vegetation Dynamics in Xinjiang, China. Remote Sens..

[9] Marian M., Licciardello G., Vicelli B., Pertot I., Perazzolli M. (2022). Ecology and potential functions of plant-associated microbial communities in cold environments. FEMS Microbiol. Ecol..

[10] Jia X.J., Qin X.M., Tian X.P., Zhao Y., Yang T., Huang J. (2021). Inoculating with the microbial agents to start up the aerobic composting of mushroom residue and wood chips at low temperature. J. Environ. Chem. Eng..

[11] Miri S., Robert T., Davoodi S.M., Brar S.K., Martel R., Rouissi T., Lauzon J.M. (2023). Evaluation of scale-up effect on cold-active enzyme production and biodegradation tests using pilot-scale bioreactors and a 3D soil tank. J. Hazard Mater..

[12] Tian X.P., Qin X.M., Jia X.J., Lyu Q.Y., Li S.Q., Jiang L.W., Chen L., Yan Z.Y., Huang J. (2024). Lignocellulose degradation and temperature adaptation mechanisms during composting of mushroom residue and wood chips at low temperature with inoculation of psychrotolerant microbial agent. Environ. Pollut..

[13] Tashiro Y., Matsumoto H., Miyamoto H., Okugawa Y., Pramod P., Miyamoto H., Sakai K. (2013). A novel production process for optically pure L-lactic acid from kitchen refuse using a bacterial consortium at high temperatures. Bioresour. Technol..

[14] Borker S.S., Thakur A., Pandey K.K., Sharma P., Manyapu V., Khatri A., Kumar R. (2024). Nutrient recycling of source-separated human faeces using biochar immobilized indigenous psychrotrophic bacteria for sustaining the agroecosystems of north-western Himalaya. Appl. Biol. Chem..

[15] Cruz G. (2013). Boric Acid in Kjeldahl Analysis. J. Chem. Educ..

[16] Shanthi K., Balasubramanian N. (1996). Method for sampling and analysis of hydrogen sulfide. Analyst.

[17] Lin H.Y., Jiao H.C., Lin H., Xu X.G. (2025). The Evolution of Policies for the Resource Utilization of Livestock Manure in China. Agriculture.

[18] Fan H., Li C., Zhang W., Liu C., Abass O.K., Liu L., Huang X., Sun Y., Wang H., Gesiye M.W. (2025). Evaluation of pollution potential in swine manure across growth stages: Impact of dietary nutrients and management strategies. Sci. Total Env..

[19] Kumar P., Tiwari S., Uguz S., Li Z.G., Gonzalez J., Wei L., Samuel R.S., Zhang Y.H., Yang X.F. (2024). Bioaerosols downwind from animal feeding operations: A comprehensive review. J. Hazard. Mater..

[20] Hu J.Y., Han X.Y., Chen H.P., Li J.Z., Wang Z.L., Luo X.C., Deng J.J. (2025). Biodegradation of skatole by Bacillus subtilis GDAAS-A32 and its inhibition for odor emissions from swine manure. J. Environ. Chem. Eng..

[21] D’Amico S., Collins T., Marx J.C., Feller G., Gerday C. (2006). Psychrophilic microorganisms: Challenges for life. EMBO Rep..

[22] Lay C.Y., Mykytczuk N.C.S., Yergeau É., Lamarche-Gagnon G., Greer C.W., Whyte L.G. (2013). Defining the Functional Potential and Active Community Members of a Sediment Microbial Community in a High-Arctic Hypersaline Subzero Spring. Appl. Environ. Microbiol..

[23] Li C.N., Zhang C., Ran F., Yao T., Lan X.J., Li H.Y., Bai J., Lei Y., Zhou Z., Cui X.N. (2024). Effects of microbial deodorizer on pig feces fermentation and the underlying deodorizing mechanism. Waste Manag..

[24] Zhang C.F., Zheng L.X., Zhang Q.X., Zhang Y.X., Zheng X.Y. (2026). Synergistic removal of methanethiol and other odorant gases by a metabolically complementary synthetic consortia isolated from food waste. Bioresour. Technol..

[25] Zhu Z.Q., Yang Y., Fang A.R., Lou Y., Xie G.J., Ren N.Q., Xing D.F. (2020). Quorum sensing systems regulate heterotrophic nitrification-aerobic denitrification by changing the activity of nitrogen-cycling enzymes. Environ. Sci. Ecotechnol..

[26] Wang J.L., Huan C.C., Lyu Q., Tian X.P., Liu Y., Ji G.S., Yan Z.Y. (2025). Efficacy of composite bacterial deodorant constructed with Camellia sinensis and its in-situ deodorization mechanism on pig manure. Waste Manag..

[27] Njalam’mano J.B.J., Chirwa E.M.N., Seabi R.L. (2020). In Vitro Study of Butyric Acid Deodorization Potential by Indigenously Constructed Bacterial Consortia and Pure Cultures from Pit Latrine Fecal Sludge. Sustainability.

[28] Fibriana F., Bahatmaka A., Imani N.A.C., Kusumaningtyas R.D., Juliadi I.A., Pradana B.A., Wafiroh H. (2025). Innovative Bio Ball System: Immobilized Rhizopus oligosporus F-05 for Effective Odor and Color Removal in Pharmaceutical Industry Wastewater. Water Air Soil Pollut..

[29] Lyu Q., Feng Z.Z., Liu Y., Wang J.L., Xu L.S., Tian X.P., Yan Z.Y., Ji G.S. (2024). Analysis of latrine fecal odor release pattern and the deodorization with composited microbial agent. Waste Manag..

